# Risk factors of mental disorders among empty and non-empty nesters in Shanxi, China: a cross-sectional study

**DOI:** 10.1186/s12955-019-1088-y

**Published:** 2019-01-21

**Authors:** Chichen Zhang, Lihong Hou, Xiao Zheng, Ruifang Zhu, Huining Zhao, Jiao Lu, Jingmin Cheng, Xiaozhao Yousef Yang, Tingzhong Yang

**Affiliations:** 1 School of Management, Shanxi Medical University, Taiyuan, Shanxi China; 2 Center for Health Management and Policy Research, Shanxi Medical University, Taiyuan, Shanxi China; 3 Zhejiang University School of Medicine, Hangzhou, Zhejiang China; 4 School of Nursing, Shanxi Medical University, Taiyuan, Shanxi China; 5 Department of Political Science and Sociology, Murray State University, Murray, KY USA; 6 Center for Tobacco Control Research, Zhejiang University School of Medicine, Hangzhou, Zhejiang China

**Keywords:** Mental disorders, Risk factors, Elderly, Cross sectional survey

## Abstract

**Background:**

China has the world’s largest size of elderly population. Mental disorders of the elders deserve keen attention. This study aims to comparatively explore mental disorders and risk factors among empty and non-empty nesters.

**Methods:**

Using the stratified random cluster sampling method, we recruited 8526 elders aged 60 years and above from 11 cities in Shanxi Province in central China, comprising 4901 empty and 3625 non-empty nesters. The mental disorders were assessed with the 12-item Chinese Health Questionnaire (CHQ-12). The binary logistic regression was performed to analyze the risk factors in the two groups.

**Results:**

The prevalence of mental disorders in empty nesters was significantly higher than that in non-empty ones (26.9% vs 23.5%). Binary logistic regression showed that the empty nesters who lived alone or lived in an institution were more likely to suffer from mental disorders compared with the non-empty ones, empty nesters living with spouse showed no significant deviation. And single status, hardly or part self-care ability, having chronic diseases, having lower income, and paying less attention to daily healthy diet had positive associations with mental disorders for empty nesters, and no or partial self-care ability and having chronic diseases for non-empty nesters.

**Conclusions:**

The study indicated that empty nesters had a higher prevalence of mental disorders than non-empty ones. Empty nesters living alone, with no or partial self-care ability, chronic disease, lower income and less attention to daily healthy diet were more vulnerable to mental disorders. And the lack of self-care ability and having chronic diseases were risk factors of mental disorders for non-empty nesters.

## Background

China has a rapidly aging population. According to the annual data of the National Bureau of Statistics of China (CNBS), 230 million people aged 60 years and above accounted for 16.7% of the total population in 2016, an increase of 15.6% from 199 million in 2010 [1, 2]. Moreover, traditional living arrangement has been challenged by the disintegration of the extended family in recent years. With the decline in the number of adult children available to help the needing elders, the proportion of elders living alone or living with their spouse is increasing. These elders are called the “empty nesters” [3]. The estimated number of empty nester households will account for 90% of the elderly households by 2030 [4].

As substantial demographic change happens, mental disorders (e.g., depression, anxiety, impulse control, and substance abuse) in the elders become a type of major health challenge [5, 6]. Among the consequences of mental disorders are loneliness, increased risk for physical illnesses (e.g., ischemic heart disease, stroke, cancer), cognitive decline, impairments in activities of daily living. And depression, a widespread type of mental disorders, causes a common disability in the elders and decreases in life satisfaction [5]. In addition, the incidence of dementia has increased at an alarming rate worldwide, especially in China where over nine million elders suffer from dementia, constituting 3.2 to 9.9 percentage of the Chinese elders [6]. In addition, Mental disorders play a prime role for suicide attempt in rural China [7] and almost all the elderly suicide victims are preceded by mental disorders [8]. Taking care of elders with mental disorders brings considerable physical, mental and financial spending to their families, and needs substantial of medical resources and healthcare support from government assistance, the community and institution care [6]. Hence, the primary prevention of mental disorders and promotion of mental health of the elderly are of utmost importance [9], and an evaluation of mental disorders and its risk factors among elders is urgently needed.

Previous studies showed that empty nesters without a traditional family providing for them were in a dire condition regarding life satisfaction [10], quality of life [11], health-promoting lifestyle [4] and social support [12]. Does the empty-nest living arrangement contribute to the mental disorders of elders? There have been some researches to report the mental disorders of elders or empty nesters. Yu [13] indicated that over 39% of elders reported depressive symptoms, which were related with the lack of social support and poor physical health. A researcher declared that the Geriatrics Depression Scale score, which was adopted to assessed the level of depression, was significantly higher in rural empty nesters. A cross-sectional study from Sichuan province showed that anxiety disorders prevailed in empty nesters [14]. The associations with mental disorders were found in all aspects of gender, relationship with their children, monthly income, chronic diseases and self-care ability [15–17]. However, studies focusing on the disparity of mental disorders and its related factors among empty and non-empty nesters were relatively less. Several researches indicated that empty nesters had higher levels regarding depressive symptoms and loneliness feelings compared with non-empty ones [18–20], while our pilot study (*n* = 488) conducted in Taiyuan, capital of Shanxi Province, China, suggested that non-empty nesters had poorer mental health status compared with empty ones, such as in somatization, obsessive-compulsiveness and psychoticism [21]. Based above, we expanded the research areas and increased the number of people surveyed to explore which elderly group was more susceptible to mental disorders. In addition, the pilot study used Symptom Check-list 90 (SCL-90), an international self-assessment questionnaire of psychological problems and symptom distress, as the survey tool [21, 22]. But in the process of actual investigation, we found that the compliance and acceptance of the elders was poor due to the more entries of SCL-90. In this study, we adopted Chinese Health Questionnaire (CHQ-12) as the research tool for measuring mental disorders, which has been suggested as effective as and more efficient than the longer version [23, 24], and also seems to be more suitable for elders than the longer version due to its simplicity. The purpose of this study was to comparatively evaluate the mental disorders among empty and non-empty nesters, and explore the risk factors among empty and non-empty nesters, thus providing effective theoretical basis for interventions and prevention of the mental disorders among empty and non-empty nesters.

## Methods

### Design and participants

The selected survey site was Shanxi Province in Central China. Using the stratified random cluster sampling method, we recruited the elderly (aged 60 years and above) from Shanxi Province’s 11 cities, namely, Taiyuan, Datong, Yangquan, Changzhi, Jincheng, Shuozhou, Jinzhong, Yuncheng, Xinzhou, Linfen, and Lvliang. The sampling method is as follows: in the first stage, we numbered the districts (counties) of 11 cities with the order of subordinate municipal districts (counties) in the government websites. In the second stage, a random number table was employed to select two districts (counties) in each city, and then two communities (administrative villages) were drawn from each district (county) in the same way. In the fourth stage, we extracted two residential areas (villages) from each community (administrative village). The elders in the two residential areas (natural villages) who met the criteria were included in this study. The inclusion criteria were (1) willingness to participate in the investigation; (2) being aged 60 years and above; and (3) having normal cognitive function. Meanwhile, elders who were diagnosed with cognitive disorders or serious illnesses, were excluded.

All participants were informed of the purpose and procedure of the research upon their recruitment, and assured of their right to refuse to participate. Their anonymity and confidentiality were guaranteed. The elders with poor understanding were consented by their children. Ethical approval for all the study procedures was sought and received from the Ethics Committee of Shanxi Medical University.

The study was conducted from June 2016 to July 2017, involving 9000 elders, of which 8526 completed the questionnaire. The response rate was 94.7%.

### Assessments and procedure

A face-to-face questionnaire interview was administered to collect information. Elders were interviewed by research assistants, who were psychology graduate students and had received lengthy and rigorous training in professional interview skills to complete the questionnaire. The questionnaire comprised two sections: the self-made basic information section and CHQ-12.

The self-made basic information questionnaire was used to measure sociodemographic characteristics, including gender, age, marital status, educational level, employment status, monthly income, residence, relationship with children, attention to health diet or not, self-care ability, and chronic diseases. Moreover, the information of chronic diseases was collected through self-reporting, which was based on the diagnostic evidence of medical records or prescriptions from doctors.

We employed the CHQ-12 to evaluate mental disorders. The CHQ-12 is a psychiatric screening instrument, which was developed by discriminant function analysis on the 30-item CHQ that is a Chinese translation version of the General Health Questionnaire (GHQ) originally developed by Goldberg with addition of several culturally-relevant items [25, 26]. The CHQ-12 of unidimensional scale has been proved to have good reliability and validity for mental disorders screening in community population or among primary health care patients in mainland China (Cronbach’α was 0.75, and except for question 7 and 10, each item had high load value for all > 0.50) (*n* = 1249) [23, 27]. The CHQ-12 includes 12 items with responses on a 4-point Likert scale (0 = “not at all,” 0 = “same as usual,” 1 = “rather more than usual,” and 1 = “much more than usual”). Questions 7 and 10 involve reverse scores. The total score is obtained by summing up the scores of the 12 items, and is used as an indicator of the analysis to explore mental disorders. A score of 4 represents the cut-point, and ≥ 4 indicates higher mental disorders [23, 28]. In this surveyed population, the internal consistency of the questionnaire was high, overall Cronbach’s α = 0.838, and the one-dimensional scale’s twelve load values were 01(0.67), 02(0.75), 03(0.77), 04(0.72), 05(0.62), 06(0.63), 07(0.33), 08(0.66), 09(0.71), 10(0.28), 11(0.55), 12(0.62), meaning that except for question7 and 10, the rest were all > 0.55.

In the procedure, a research assistant read and explained the contents of the questionnaire to a participant using a standardized guide. After obtaining the participant’s answer, the research assistant provided a blank questionnaire for the participant to fill out and then retrieved it on the spot.

### Statistical analysis

Basic information was shown as numbers (percentages) using the descriptive analysis. The chi-square test was employed to compare the differences in basic characteristics among empty and non-empty nesters. The one-way ANOVA and independent sample t-test were used to compare the differences between the basic characteristics and their mental disorders. The binary logistic regressions were performed to evaluate the associations of different living arrangements and these characteristics related to mental disorders in the two groups, respectively. Odds ratios (ORs) and 95% confidence intervals (95% CI) for risk of mental disorders were calculated. Data analyses were carried out using SPSS version 24.0 for windows. *P*-values lower than 0.05 were considered statistically significant.

## Results

### Basic characteristics

A total of 8526 elders were recruited in our study, comprising 4901 (57.5%) empty and 3625 (42.5%) non-empty nesters. The mean age was 70.2 ± 6.85 years old. Among the participants, 4239 were males (49.7%) and 4290 were females (50.3%). More than half of them lived in rural areas (65.01%), obtained elementary education and below (62.8%), and had either no monthly income or income less than RMB 1000 (62.5%). About three-fourths were married (70.8%). And 81.0% of them could care for themselves completely. Statistically significant differences in basic characteristics among empty and non-empty nesters were found for gender, age, marital status, employment status, monthly income, chronic disease, attention to daily healthy diet, relationship with their children and CHQ-12 score. Among the participants, 1317 empty (26.9%) and 853 (23.5%) non-empty nesters had a total score of 4 and above. Thus, the prevalence of mental disorders was 25.5% in all participants; it was higher in the empty elders than in non-empty ones (26.9% vs. 23.5%) (*P* < 0.05; Table 1).

**Table 1:** Comparison of the characteristics and mental disorders among empty nesters and non-empty ones

Characteristics	Empty nest [n(%)]	Non-empty nest [n(%)]	χ^2^	*P*-value
Gender			22.932	< 0.001
Male	2546 (51.9)	1693 (46.7)		
Female	2355 (48.1)	1932 (53.3)		
Age			95.133	< 0.001
Low (60–69 years)	2440 (49.8)	2130 (58.8)		
Middle (70-79 years)	1910 (39.0)	1046 (28.9)		
High(80 years or above)	551 (11.2)	449 (12.4)		
Education status			2.057	0.358
Elementary education and below	3073 (62.7)	2279 (62.9)		
Secondary education	1599 (32.6)	1154 (31.8)		
Higher education and above	229 (4.7)	192 (5.3)		
Marital status			39.495	< 0.001
Single(never married, divorced, widowed)	1560 (31.8)	927 (25.6)		
Married	3341 (68.2)	2698 (74.4)		
Employment status			20.175	< 0.001
Working	516 (10.5)	278 (7.7)		
Not working	4385 (89.5)	3347 (92.3)		
Monthly income			22.174	< 0.001
No income	1676 (34.2)	1384 (38.2)		
Low (< 1000 RMB)	1371 (28.0)	898 (24.8)		
Middle (1000–3000 RMB)	1287 (26.3)	884 (24.4)		
High (> 3000 RMB)	567 (11.6)	459 (12.7)		
Residence			0.001	0.974
Rural	3187 (65.0)	2356 (65.0)		
Urban	1714 (35.0)	1269 (35.0)		
Chronic disease			51.486	< 0.001
Yes	1440 (29.4)	1332 (36.7)		
No	3461 (70.6)	2293 (63.3)		
Attention the daily healthy diet			10.919	0.012
No	152 (3.1)	75 (2.1)		
Less	1326 (27.1)	954 (26.3)		
more	2547 (52.0)	1964 (54.2)		
Most	876 (17.9)	632 (17.4)		
Relationship with their children			262.368	< 0.001
Bad	858 (17.5)	209 (5.8)		
Good	4043 (82.5)	3416 (94.2)		
Self-care ability			0.488	0.485
Hardly or part	921 (18.8)	703 (19.4)		
Completely	3980 (81.2)	2922 (80.6)		
Living arrangement			–	–
Living with children	–	3625 (100)		
Living with spouse	3035 (61.9)	–		
Living alone	1651 (33.7)	–		
Living in an institution	215 (4.4)	–		
Mental disorders			12.259	< 0.001
Yes	1317 (26.9)	853 (23.5)		
No	3584 (73.1)	2772 (76.5)		

### Associations with mental disorders in different living arrangements

According to different living arrangements, we further divided empty nest into three living patterns: living with spouse, living alone and living in an institution. The results demonstrated that the empty nesters who lived alone or lived in an institution were more likely to suffer from mental disorders compared with the non-empty ones (living with children) in both basic and adjusted model, the OR was 1.49 (1.30, 1.70) and 1.38 (1.02, 1.86), respectively, whereas it was not prominent in the empty nesters who lived with spouse (*P* < 0.05; Table 2).

**Table 2:** Empty and non-empty nesters in different arrangements with mental disorders

Living pattern	Crude OR	95% CI	Adjusted OR	95% CI
Non-empty nest				
Living with children	Reference	–	Reference	–
Empty nest				
Living with spouse	0.99	0.88, 1.11	1.07	0.96, 1.21
Living alone	1.57**	1.38, 1.78	1.49**	1.30, 1.70
Living in an institution	1.60*	1.19, 2.15	1.38*	1.02, 1.86

Note: Adjusted for gender, age, marital status, educational level, employment status, monthly income, residence, relationship with children, attention to health diet or not, self-care ability, and chronic diseases

*Significant at *P* < 0.05

**Significant at *P* < 0.001

### Related risk factors with mental disorders

*P*-values in Table 3 demonstrated that, except for gender, age, employment status and relationship with their children, the CHQ-12 scores for empty nesters across different demographic characteristics had statistically significant differences. And there were statistically significant differences in CHQ-12 scores with respects to age, monthly income, chronic disease, and self-care ability among non-empty nesters. In addition, significant differences of CHQ-12 scores among empty and non-empty nesters in the same characteristic were shown in female, middle age (70~79 years), elementary education, married status, employment status, no income, middle income (1000~3000RMB), living in urban, having no chronic disease, paying no or less attention to daily diet, having a good relationship with their children and no or partial self-care. The CHQ-12 scores in empty nesters were all higher compared with those in non-empty ones, in addition to middle income (*P* < 0.05; Table 3).

**Table 3:** CHQ-12 scores by characteristics among empty and non-empty nesters

Characteristics	Empty nesters			Non-empty nesters		
	Mean	F/*t*-value^a^	*P*-value^a^	Mean	F/*t*-value^b^	*P*-value^b^
Gender		0.834	0.337		0.026	0.654
Male	2.98 ± 1.67			2.88 ± 1.65		
Female	3.03 ± 1.72			2.86 ± 1.63*		
Age		0.689	0.502		3.358	0.035
Low (60–69 years)	2.98 ± 1.68			2.91 ± 1.67		
Middle (70-79 years)	3.02 ± 1.71			2.76 ± 1.57**		
High (80 years or above)	3.07 ± 1.77			2.91 ± 1.64		
Education status		14.568	< 0.001		2.824	0.060
Elementary education	3.10 ± 1.73			2.91 ± 1.67*		
Secondary education	2.87 ± 1.65			2.81 ± 1.63		
Higher education	2.66 ± 1.51			2.67 ± 1.32		
Marital status		38.401	< 0.001		6.04	0.090
Single (never married, divorced, widowed)	3.19 ± 1.81			2.95 ± 1.70		
Married	2.92 ± 1.64			2.84 ± 1.62*		
Employment status		4.087	0.308		2.953	0.253
Working	3.01 ± 1.72			2.88 ± 1.65**		
Not working	2.93 ± 1.56			2.76 ± 1.56		
Monthly income		24.03	< 0.001		4.56	0.003
No income	3.20 ± 1.79			2.84 ± 1.61**		
Low (< 1000 RMB)	3.12 ± 1.79			2.94 ± 1.73		
Middle (1000–3000 RMB)	2.80 ± 1.52			2.95 ± 1.71*		
High (> 3000 RMB)	2.64 ± 1.47			2.64 ± 1.37		
Residence		23.64	< 0.001		0.678	0.393
Rural	3.08 ± 1.76			2.88 ± 1.66		
Urban	2.86 ± 1.59			2.84 ± 1.61**		
Chronic disease		15.553	< 0.001		36.064	< 0.001
Yes	3.17 ± 1.78			3.09 ± 1.82		
No	2.94 ± 1.66			2.74 ± 1.52**		
Attention to daily healthy diet		31.771	< 0.001		0.163	0.921
No	3.78 ± 2.20			2.85 ± 1.78*		
Less	3.29 ± 1.88			2.88 ± 1.63**		
more	2.90 ± 1.59			2.85 ± 1.62		
Most	2.79 ± 1.54			2.89 ± 1.71		
Relationship with their children		0.011	0.605		2.307	0.149
Bad	3.03 ± 1.72			2.71 ± 1.66		
Good	3.00 ± 1.70			2.88 ± 1.64**		
Self-care ability		233.258	< 0.001		186.048	< 0.001
Hardly or part	3.69 ± 2.14			3.59 ± 2.05*		
Completely	2.85 ± 1.54			2.69 ± 1.48		

Note: ^a^ and ^b^ were the statistical outcomes of ANOVA and t-test for CHQ-12 scores of empty nesters (non-empty nesters) in different basic characteristics

The symbol “*” (“**”) indicated that the statistical level was *P* < 0.05 (*P* < 0.001) for the comparison of CHQ-12 scores among empty and non-empty nesters in the same characteristic

To explore the associations with mental disorders for empty nesters and non-empty ones, we used the sociodemographic variables that had statistically significant differences in one-way ANOVA for empty nesters (non-empty ones) as covariates, and then set the last item of each variable as a sub-variable. The covariates for empty nesters included education, marital status, monthly income, residence, chronic disease, attention to daily healthy diet and self-care ability. For non-empty nesters, the covariates were age, monthly income, chronic disease and self-care ability. The CHQ-12 total scores were defined as the dependent variable in the logistic regression analysis. We used the method of Forward: LR, with inclusion and exclusion criteria defined as 0.05 and 0.10, respectively. Then logistic regression models for empty and non-empty nesters were established. Hosmer-Lemeshow tests in both two groups all showed good fits for the two regression models (*P* = 0.974 > 0.05; *P* = 0.677 > 0.05).

Based on the results of the logistic regression analysis, single status (never married, divorced, or widowed) (OR = 1.22; 95% CI = 1.06, 1.40); no or partial self-care ability (OR = 2.31; 95% CI = 1.98, 2.69); having chronic diseases (OR = 1.28; 95% CI = 1.11, 1.47); having no monthly income (OR = 1.76; 95% CI = 1.38, 2.26) or monthly income less than RMB 1000 (OR = 1.67; 95% CI = 1.30, 2.16) or between RMB 1000 and 3000 (OR = 1.36; 95% CI = 1.05, 1.76), and never paying attention to daily healthy diet (OR = 2.04; 95% CI = 1.41, 2.97) or paying less attention (OR = 1.58; 95% CI = 1.29, 1.94) had positive connections with mental disorders for empty-nesters. The lack of self-care ability (OR = 2.78; 95% CI = 2.32, 3.32) and having chronic diseases (OR = 1.25; 95% CI = 1.06, 1.47) were positively associated with mental disorders for non-empty elders. And middle age (70~79 years) (OR = 0.753; 95% CI = 0.58, 0.98) was a protective factor for non-empty ones (Table 4).

**Table 4:** Results of logistic regression for association of characteristics with mental disorders

Variable	Empty nest		Non-empty nest	
	Adjusted OR	95% CI	Adjusted OR	95% CI
Age				
High (80 years or above)	–		1.00	
Middle (70–79 years)	–	–	0.753*	0.58, 0.98
Low (60–69 years)	–	–	1.010	0.79, 1.28
Self-care ability				
Completely	1.00		1.00	
Hardly or part	2.31**	1.98, 2.69	2.78**	2.32, 3.32
Chronic disease				
No	1.00		1.00	
Yes	1.28**	1.11, 1.47	1.25**	1.06, 1.47
Marital status				
Married	1.00		–	
Single (never married, divorced, widowed)	1.22*	1.06, 1.40	–	–
Monthly income				
High (>RMB 3000)	1.00		–	
Middle (RMB 1000–RMB 3000)	1.36*	1.05, 1.76	–	–
Low (<RMB 1000)	1.67**	1.30, 2.16	–	–
No income	1.76**	1.38, 2.26	–	–
Attention to daily healthy diet				
Most	1.00		–	
More	1.07	0.88, 1.29	–	–
Less	1.58**	1.29, 1.94	–	–
No	2.04**	1.41, 2.97	–	–

Note: Adjusted for educational status, marital status, monthly income, residence, chronic disease, attention to daily healthy diet, and self-care ability among empty nesters. Adjusted for age, monthly income, chronic disease, and self-care ability among non-empty nesters

*Significant at *P* < 0.05

**Significant at *P* < 0.001

## Discussion

The observed rate of mental disorders was higher in empty nesters (26.9%) compared with non-empty ones (23.5%), indicating that the former were more vulnerable to mental disorders than the latter. Moreover, the final result also supported the related comparative studies [18–20], which indicated that empty nesters had higher levels regarding specific psychiatric problem and symptom distress. However, the conclusive point was inconsistent with the findings in our pilot study [21]. Different living areas may account for the varied outcome. 65% of the participants in this study resided in rural areas, while 66.0% of the study subjects lived in urban areas in our pilot study. Many previous studies have indicated that empty nesters living in the rural had significantly more serious mental distress compared with those who lived in the city [14, 29]. In rural China, the traditional concept of ‘bring up their children for old age’ of elders is relatively strong. The elders are accustomed to relying on their children, while leaving away from their children (an empty-nest status) easily make them into a boring and hopeless life. Whereas for empty nesters living in the urban areas, their independent economic ability and open mind, as well as free time enable them to enjoy interactive activities and light mood. In addition, different measurement tools may also be responsible for the discrepancy. SCL-90 contains nine dimensions and focuses on nine aspects of mental health: somatization, obsessive-compulsive, interpersonal sensitivity, depression, anxiety, hostility, phobic anxiety, paranoid ideation and psychoticism [22], while CHQ-12 only pays attention to mental disorders in a unidimensional perspective.

After further analysis, a significant association between empty nesters who lived alone or lived in an institution and mental disorders was found. A previous study pointed that pension agency may block the social relationship network of the community where the elders were located, making elders lack necessary interactions and communications, thereby causing poorer mental health [30]. However, empty nesters living with spouse didn’t have higher mental disorders in comparison to non-empty ones. The finding reflected that the spouse had a salutary effect on empty nesters’ mental health, which was consistent with the study result of Cheng [20]. After children departed from home, their spouse may be the most understanding and closest person to them and serve as the strong pillar to provide them emotional support against loneliness or other bad emotion [31]. Our study results pointed that empty nesters overall, especially the ones who lived alone or lived in an institution, had a higher prevalence of mental disorders than non-empty nesters.

The common risk factors related to mental disorders in the two groups were in the lack of self-care ability and having chronic disease. Lower monthly income, paying no or less attention to healthy diet and single status were uniquely associated with higher mental disorders in empty nesters. And middle age(70~79 years) was a protective factor of mental disorders for non-empty nesters relative to high age (80 years or above). Moreover, chronic diseases and monthly income, which had been found and fully explained in our pilot study [21], were confirmed again in this study.

The lack of self-care ability was a risk factor contributing to mental disorders in both groups. There is a strong link between physical and mental health [32]. A mediation analysis demonstrated that physical activity is the pathway through which physical health influences mental health [33]. Community health service centers should establish various forms of fitness equipment to attract the elderly to exercise; carry out community health education and psychological counseling to elevate elders’ awareness of self-care; and improve disease rehabilitation services by strengthening specialized training of family caregivers to improve the status of hardly or part self-care ability, and to promote rehabilitation of patients’ psychological, physical, and social functions.

Being single was a risk factor in the mental disorders among empty nesters, which proved the importance of spouse who could be responsible for companionship and emotional comfort. Whereas for the non-empty nesters, the companionship of the children made up for the lack of spousal companionship, which made the impact of being single on their mental health less prominent. Therefore, married elders, especially empty nesters, should be encouraged to cherish the time spent with their old partners. And the single empty nesters should socialize with others in diverse team activities and believe a late-life love is not all they need for a good life. Simultaneously, the community-based primary prevention for mental disorders should be strengthened to promote mental health of the empty nesters, especially for single ones. In addition, adult children should increase the amount of time they spend with their parents and visit their aging parents more frequently.

In this study, the empty nesters who never attended to daily healthy diet (3.78 ± 2.20) or paid less attention (3.29 ± 1.88) had higher CHQ-12 scores than those paying more attention (2.90 ± 1.59) or most attention (2.79 ± 1.54). And never paying attention to healthy diet (OR = 2.04; 95% CI = 1.41, 2.97) or paying less attention (OR = 1.58; 95% CI = 1.29, 1.94) was significantly associated with mental disorders in empty nesters, but it was not the influencing factor for the non-empty nesters. There is a growing priority internationally about that dietary risk factor, as well as physical health, has a close relationship with increased prevalence of mental disorders [34, 35]. Previous studies presented various evidences, indicating that key nutrients or diet components, such as antioxidants, omega-3 fatty acids, and B vitamins, from a healthy whole food diet played a pivotal role in cognitive function and mental disorders [35–37]. Whereas contextual factor (social, physical, and environmental) affected dietary intake and quality [37]. In this study, contextual factors of the two subjects: empty and non-empty nesters, were different, such as family support and dietary pattern. In China, non-empty nesters living with their children have two conditions with respect to diet. One is that their children intimately arrange daily routines and reasonable diet for them; the other is that they have to prepare regular and balanced diet for their children who go to school or work. Hence, whether or not they actively focus on healthy diet, their diet is always in regular shape. This phenomenon is called “compulsive healthy diet”. So that the effect of whether or not paying attention to daily diet on mental disorders in non-empty nesters is insignificant. In contrast, empty nesters living independently have no children’s care and do not need to take care of their children. Hence, their diet depends completely on whether or not they pay attention. The community service centers should hire dietitians and food hygiene supervisors for carrying out diet health education to the elderly, in reference to the Chinese Dietary Guidelines (2016) [38]. This is especially important for empty nesters who should be instructed individually and consistently to enhance their balanced diet awareness. Health management centers should consider bringing meals-on-wheels service to empty nesters.

In addition, lower income had a positive association with mental disorders for empty nesters, which was consistent with Wang’s findings [39]. Higher income is significantly associated with lower psychological pressure, which is responsible for the marked improvement in mental health status. In rural China, the coverage of social security funds is lower than in the cities, the elder’s economic source of life is mainly from farming or family support. While non-empty nesters could depend on their children, empty nesters’ incomes are the most direct dependence for the security of life. The government should expand the coverage of welfare (especially the pension plan) [40] and give commercial pension insurance active guidance to exert its complementary role on social security of elders. Moreover, individuals should strengthen personal awareness of commercial pension insurance investment for future security. In addition, filial piety as a basic Chinese principle should be advocated; no matter where the children go to, it is incumbent for the elders.

We acknowledge the study had several limitations. First, the sample population were selected from Shanxi Province, thus these results may not be representative of the elders in other areas in China with different economic and geographical features. Second, the data collection, which was based on questionnaires and had no objective measurement, was subjective. Third, this was a cross-sectional study; therefore, the results failed to prove a causal relationship between risk factors and mental disorders.

## Conclusion

This study indicated that empty nesters, especially the ones living alone and living in an institution, were more vulnerable to mental disorders than non-empty ones. And the elderly, who could completely care themselves and did not have chronic diseases, had lower prevalence of mental disorders. While having a higher income, married status and paying more attention to daily healthy diet uniquely contributed to empty nesters’ mental disorders. The results suggested scientific evidence for targeting interventions addressing risk factors of mental disorders among elders. Moreover, it contributed to the primary prevention of mental disorders and improvement of quality of life, as well as the formulation of health management strategies for elders.

## Abbreviations

CHQ-12: The 12-item version of Chinese Health Questionnaire
SCL-90: Symptom Check-list 90
